# Independent effects of the glucose-to-glycated hemoglobin ratio on mortality in critically ill patients with atrial fibrillation

**DOI:** 10.1186/s13098-024-01401-0

**Published:** 2024-07-22

**Authors:** Yuqing Fu, Xing Wei, Cong Xu, Guifu Wu

**Affiliations:** 1 Department of Cardiology, The Eighth Affiliated Hospital, Sun Yat-Sen University, Shenzhen, 518000 Guangdong China; 2 Department of Cardiology, The Second People’s Hospital of Hefei, Hefei Hospital Affiliated to Anhui Medical University, Hefei, 230011 Anhui China

**Keywords:** Atrial fibrillation, Intensive care unit, Glucose-to-glycated hemoglobin ratio

## Abstract

**Background:**

The glucose-to-glycated hemoglobin ratio (GAR) represents stress hyperglycemia, which has been closely associated with adverse outcomes in cardio-cerebrovascular diseases. No studies have examined the association between stress hyperglycemia and atrial fibrillation (AF) in critically ill patients. This study aims to explore the relationship between GAR and the prognosis of critically ill patients with AF.

**Methods:**

A retrospective cohort of patients was selected from the Medical Information Mart for Intensive Care IV (MIMIC-IV) database. The GAR was calculated based on fasting blood glucose and glycated hemoglobin levels measured after admission. The primary outcome was the 30-day mortality rate, with secondary outcomes being the 90-day and 365-day mortality rates. The GAR was divided into tertiles, and Kaplan–Meier analysis was employed to compare differences in mortality rates between groups. The Cox proportional hazards model and restricted cubic splines (RCS) were utilized to evaluate the relationship between the GAR and mortality. Subsequently, a segmented regression model was constructed to analyze threshold effects in cases where nonlinear relationships were determined.

**Results:**

In this cohort, the second tertile of the GAR exhibited lower mortality rates at 30 days (10.56% vs 6.33% vs 14.51%), 90 days (17.11% vs 10.09% vs 17.88%), and 365 days (25.30% vs 16.15% vs 22.72%). In the third tertile, the risk of mortality at 30 days increased by 165% (HR = 2.65, 95% CI 1.99–3.54, p < 0.001), at 90 days increased by 113% (HR = 2.13, 95% CI 1.68–2.70, *p* < 0.001), and at 365 days increased by 70% (HR = 1.70, 95% CI 1.68–2.70, *p* < 0.001). The association between the GAR and patient mortality demonstrated a “J-shaped” non-linear correlation. Once the GAR exceeded 15.915, each incremental unit increase in the ratio was associated with a 27.2% increase in the risk of 30-day mortality in critically ill atrial fibrillation patients (HR = 1.262, 95% CI 1.214–1.333, *p* < 0.001).

**Conclusion:**

The GAR is associated with both short-term and long-term mortality in critically ill patients with AF in a J-shaped relationship. Both low and excessively high GAR values indicate poor prognosis.

## Introduction

AF is the most common cardiac arrhythmia worldwide, associated with increased risks of heart failure, myocardial infarction, and stroke, consequently elevating the burden of mortality [1]. Critically ill patients often face the risk of new-onset AF [2], and those with either new-onset AF or pre-existing AF during Intensive Care Unit (ICU) admission have a higher mortality rate compared to patients with no history of AF [3]. However, research on adverse prognostic factors in critically ill AF patients is limited.

Stress hyperglycemia is a physiological response to a sudden clinical event that causes an increase in blood glucose levels, a common occurrence in ICU patients [4, 5], which can induce myocardial injury through multiple mechanisms, including acidosis from lactate accumulation, heightened inflammatory responses, intracellular calcium overload, and disturbances in lipid metabolism [6]. Given that the myocardium predominantly utilizes fatty acids as its energy source [7], patients with AF experience exacerbated cardiac damage due to increased myocardial glycolysis and the accumulation of late-stage glucose metabolic byproducts, which result from myocardial injury and rapid, disorganized electrical activity [8]. Meanwhile, hypoglycemia is a risk factor for cardiovascular disease and mortality, particularly among individuals with concomitant arrhythmias [9]. Evaluating the association between stress hyperglycemia and critically ill AF patients is essential. The GAR, representing the ratio of plasma glucose concentration to glycated hemoglobin (the baseline average glucose over the past 3 months), quantifies acute plasma glucose elevation. Additionally, the GAR quantifies acute plasma glucose elevation. Previous studies have linked elevated GAR indices to outcomes following ischemic stroke and thrombolytic therapy [10–12]. This study represents the inaugural assessment of the correlation between stress hyperglycemia, delineated by the GAR, and the prognosis of critically ill AF patients, thereby furnishing valuable insights for tailored glucose management strategies.

## Methods and materials

### Study population

This retrospective study extracted data on patients with AF from the MIMIC-IV database, a large database developed and managed by the Laboratory for Computational Physiology at the Massachusetts Institute of Technology. The database contains medical information on patients admitted to the intensive care units of the Beth Israel Deaconess Medical Center. The first author of this study obtained permission to access the dataset and extracted the relevant data. The use of this database for research has been approved by the institutional review boards of the Massachusetts Institute of Technology and the Beth Israel Deaconess Medical Center.

**Fig. 1:**
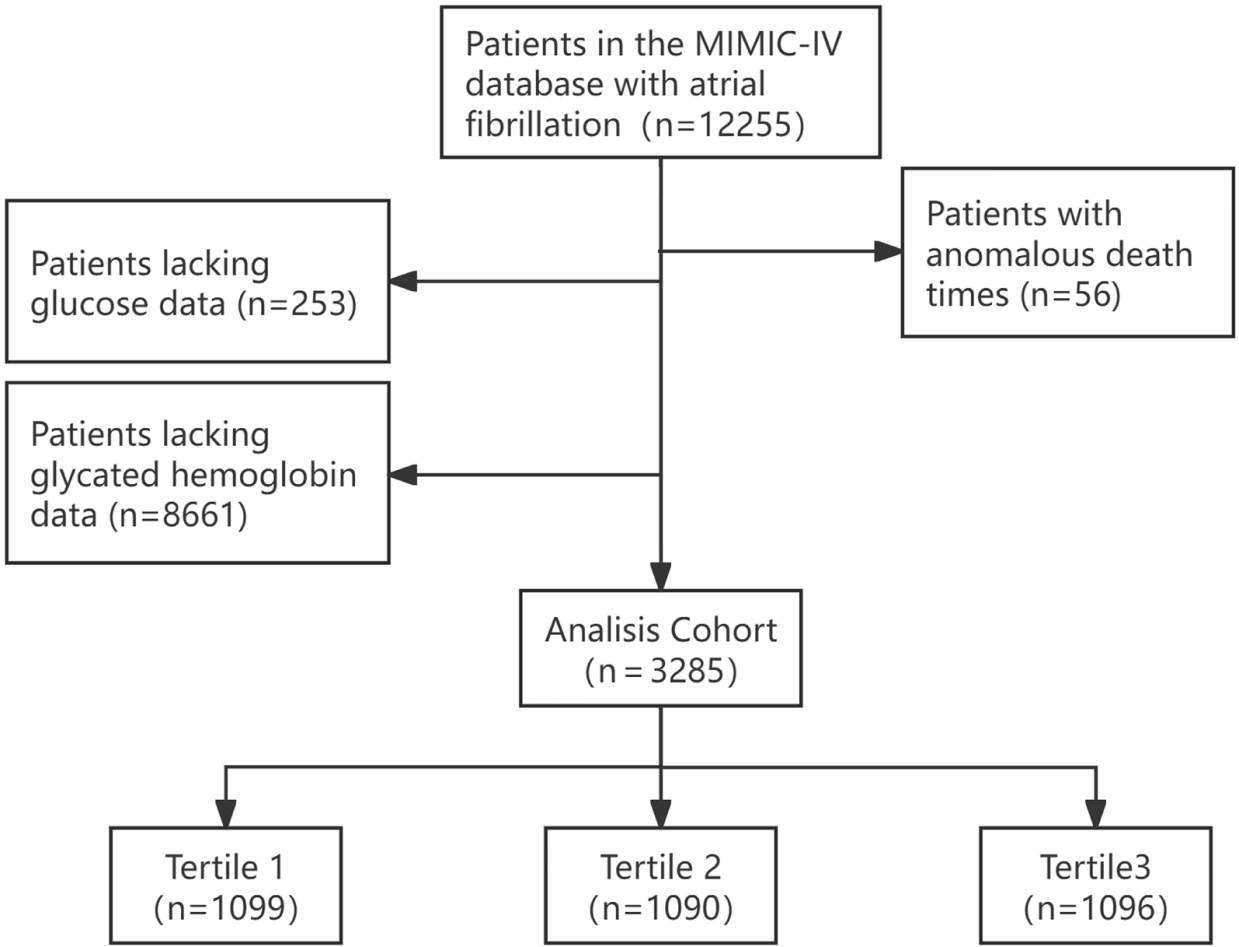
A flowchart of patient selection

In this study, 12,255 patients with AF who were admitted to the ICU for the first time were included, diagnosed according to the International Classification of Diseases, Ninth Revision (ICD-9) and Tenth Revision (ICD-10) codes. Exclusions were made for 253 cases lacking glucose data, 56 cases with anomalous death times, and 8,661 cases lacking data on glycated hemoglobin, ultimately resulting in the inclusion of 3,285 critically ill patients with AF. A flowchart of patient selection was shown as Fig. 1.

### Data extractions

PostgreSQL software (version 13.7.2) was used to extract data via Structured Query Language (SQL). Potential covariates included in this study were: (1) Baseline demographic information: age, gender, race, and body mass index (BMI). (2) Comorbidities: hypertension, diabetes, acute kidney injury (AKI), chronic kidney disease (CKD), acute myocardial infarction (AMI), heart failure (HF), stroke, cancer, and hyperlipidemia. (3) Laboratory parameters: fasting blood glucose, glycated hemoglobin(HbA1c), white blood cells (WBC), hemoglobin (HGB), serum creatinine, serum uric acid, serum lactate, international normalized ratio (INR), D-dimer, triglycerides, and low-density lipoprotein cholesterol (LDL-C). (4) Disease severity scores: Oxford Acute Severity of Illness Score (OASIS) and Sequential Organ Failure Assessment (SOFA) score. Due to more than 30% missing data for serum lipids, serum uric acid and D-dimer these were not included in the statistical analysis. Missing data for other variables included in the analysis were imputed using the random forest method for all serological indicators.

### Exposure variables

Stress hyperglycemia syndrome was estimated using the GAR, calculated by the formula: fasting blood glucose (mg/dL) / HbA1c (%). As critically ill patients in the MIMIC database do not have a separately defined fasting blood glucose, the lowest blood glucose level during hospitalization was used as a proxy for fasting blood glucose. Patients were stratified into three groups based on the tertiles of the GAR.

### Outcome events

The primary outcome of this study was all-cause mortality at 30 days following ICU admission, with secondary outcomes including all-cause mortality at 90 days and 365 days post-admission.

### Statistical analysis

For this study, categorical variables were presented as percentages, and chi-square tests were employed to evaluate the significance of differences in categorical variables among various GAR groups. Normality tests were performed for all continuous variables; non-normally distributed variables were represented by median (interquartile range) and compared using non-parametric rank-sum tests. Patients were divided into three groups based on GAR tertiles, with the second tertile serving as the reference. The Cox proportional hazards model was used to assess hazard ratio (HR) for outcome events, incorporating age, gender, race, BMI, AKI, CKD, HF, hypertension, cancer, stroke, WBC, hemoglobin, creatinine, serum lactate, SOFA score and OASIS score as confounders in the multivariate Cox regression model. AMI and diabetes did not meet the Cox proportional hazards assumption and were therefore not included in the model.

Survival analysis was conducted using the Kaplan–Meier method based on GAR tertiles, with inter-group differences assessed using the log-rank test. Restricted cubic splines (RCS) were utilized to explore the correlation between GAR and outcome events, and a threshold effect model was established to analyze the inflection points of GAR. Subgroup analyses were performed to verify the robustness of the results. Statistical analyses in this study were conducted using R Studio (version R4.2.3) and IBM SPSS Statistics (version V22.0). A two-sided *P*-value of < 0.05 was considered statistically significant.

## Results

### Patients' baseline information

The study cohort comprised 3,285 patients with critical illness and a diagnosis of AF. Mortality rates within the cohort were as follows: 344 patients (10.47%) succumbed within 30 days, 494 patients (15.04%) within 90 days, and 703 patients (21.40%) within 1 year of the initial diagnosis. The baseline characteristics patient according to tertile of GAR (1099 patients in tertile 1 [1.97–14.03]; 1090 patients in tertile 2 [14.04–16.54]; and 1096 patient in tertile 3 [16.55–40.32] are summarized in Table 1. Compared to patients in Tertile 2, those with lower and higher GAR values exhibited increased short-term and long-term mortality rates. Meanwhile, Tertile 3 had a higher proportion of diabetics than Tertile 1, but similar to Tertile 2.

**Table 1:** Patients' baseline information

Characteristic	Total (n = 3285)	Tertile1 (n = 1099)	Tertile2 (n = 1090)	Tertile3 (n = 1096)	*P*-value
Age (years)					0.939
< 65	705 (21.46)	233 (21.20)	233 (21.38)	239 (21.81)	
≥ 65	2580 (78.54)	866 (78.80)	857 (78.62)	857 (78.19)	
Gender (%)					0.068
Male	1288 (39.21)	460 (41.86)	421 (38.62)	407 (37.14)	
Female	1997 (60.79)	639 (58.14)	669 (61.38)	689 (62.86)	
Race, n (%)					0.010
White	2185 (66.51)	696 (63.33)	761 (69.82)	728 (66.42)	
Black	149 (4.54)	61 (5.55)	47 (4.31)	41 (3.74)	
Other	951 (28.95)	342 (31.12)	282 (25.87)	327 (29.84)	
BMI, kg/m2, n (%)					0.023
≤ 24.9	749 (22.8)	277 (25.2)	257 (23.6)	215 (19.6)	
25–30	1096 (33.4)	351 (31.9)	372 (34.1)	373 (34)	
> 30	1440 (43.8)	471 (42.9)	461 (42.3)	508 (46.4)	
Hypertension, n (%)					< 0.001
No	1628 (49.56)	620 (56.41)	513 (47.06)	495 (45.16)	
Yes	1657 (50.44)	479 (43.59)	577 (52.94)	601 (54.84)	
Diabetes, n (%)					< 0.001
No	2141 (65.18)	492 (44.77)	830 (76.15)	819 (74.73)	
Yes	1144 (34.82)	607 (55.23)	260 (23.85)	277 (25.27)	
Heart failure, n (%)					< 0.001
No	1857 (56.53)	523 (47.59)	636 (58.35)	698 (63.69)	
Yes	1428 (43.47)	576 (52.41)	454 (41.65)	398 (36.31)	
AMI, n (%)					0.016
No	2813 (85.63)	916 (83.35)	955 (87.61)	942 (85.95)	
Yes	472 (14.37)	183 (16.65)	135 (12.39)	154 (14.05)	
Cancer, n (%)					0.035
No	2733 (83.20)	902 (82.07)	893 (81.93)	938 (85.58)	
Yes	552 (16.80)	197 (17.93)	197 (18.07)	158 (14.42)	
CKD, n (%)					< 0.001
No	2577 (78.45)	774 (70.43)	893 (81.93)	910 (83.03)	
Yes	708 (21.55)	325 (29.57)	197 (18.07)	186 (16.97)	
AKI, n (%)					< 0.001
No	2341 (71.26)	668 (60.78)	804 (73.76)	869 (79.29)	
Yes	944 (28.74)	431 (39.22)	286 (26.24)	227 (20.71)	
Stroke, n (%)					0.007
No	2789 (84.90)	963 (87.63)	906 (83.12)	920 (83.94)	
Yes	496 (15.10)	136 (12.37)	184 (16.88)	176 (16.06)	
Hyperlipidemia, n (%)					0.960
No	1494 (45.48)	496 (45.13)	498 (45.69)	500 (45.62)	
Yes	1791 (54.52)	603 (54.87)	592 (54.31)	596 (54.38)	
HbA1c, %, M (Q₁, Q₃)	5.90 (5.50, 6.50)	6.40 (5.90,7.50)	5.80 (5.50,6.10)	5.70 (5.30,6.10)	< 0.001
Glugose, (mmol/L), M (Q₁, Q₃)	90.00 (80.00, 100.00)	75.00 (65.00,84.00)	89.00 (84.00,95.00)	102.00 (95.00,115.00)	< 0.001
WBC (× 10^9^/L), M (Q₁, Q₃)	11.10 (8.20, 14.90)	11.40 (8.20,15.40)	11.10 (8.20,14.80)	11.05 (8.30,14.60)	0.475
HGB(g/L), M (Q₁, Q₃)	104.0 (88.0, 122.0)	98.0 (85.0,116.0)	104.0 (88.0,122.0)	109.0 (91.0,126.0)	< 0.001
Creatinine, (mg/dL) M (Q₁, Q₃)	1.00 (0.80, 1.30)	1.00 (0.80,1.45)	0.90 (0.70,1.20)	0.90 (0.70,1.20)	< 0.001
serum lactate, (mmol/L) M (Q₁, Q₃)	1.8 (1.3, 2.6)	1.9 (1.3, 2.7)	1.8 (1.3, 2.7)	1.8 (1.3, 2.5)	0.048
INR, M (Q₁, Q₃)	1.40 (1.20, 1.60)	1.40 (1.20,1.60)	1.40 (1.20,1.60)	1.30 (1.20,1.50)	< 0.001
SOFA, M (Q₁, Q₃)	5.00 (3.00, 7.00)	5.00 (3.00,8.00)	4.00 (2.00,7.00)	4.00 (2.00,6.00)	< 0.001
OASIS, M (Q₁, Q₃)	32.00 (27.00, 37.00)	33.00 (27.00,39.00)	31.00 (27.00,37.00)	31.00 (26.00,37.00)	< 0.001
30-day mortality, n (%)	344 (10.47)	116 (10.56)	69 (6.33)	159 (14.51)	< 0.001
90-day mortality, n (%)	494 (15.04)	188 (17.11)	110 (10.09)	196 (17.88)	< 0.001
365-day mortality, n (%)	703 (21.40)	278 (25.30)	176 (16.15)	249 (22.72)	< 0.001

Continuous numerical variables are expressed as medians (interquartile spacing) and categorical variables are expressed as numbers (percentages). M: Median, Q₁: 1st Quartile, Q₃: 3st Quartile

*AMI* acute myocardial infarction, *CKD* chronic kidney disease, *AKI* acute kidney injury, *GAR* glucose-to-glycated hemoglobin ratio, *INR* international normalized ratio, *SOFA* sepsis-organ failure assessment score, *OASIS* Oxford acute severity of illness score, *WBC* white blood cells, *RBC* red blood cells, *HGB* hemoglobin

### Survival analysis

**Fig. 2:**
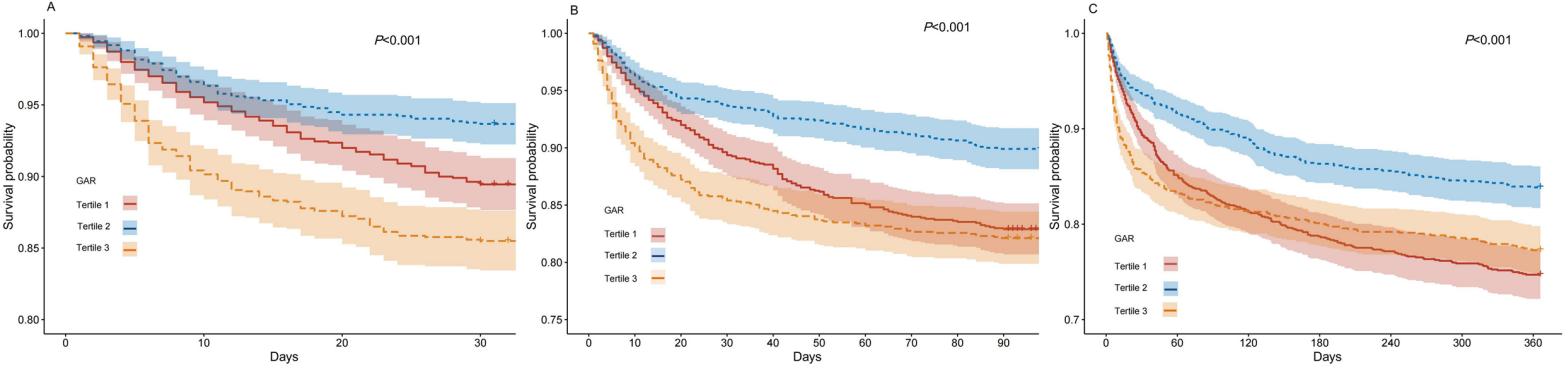
Kaplan–Meier all-cause mortality survival analysis curve. **A** Relationship between GAR tertile groups and 30-day mortality; **B** Relationship between GAR tertile groups and 90-day mortality; **C** Relationship between GAR tertile groups and 365-day mortality

Kaplan–Meier survival analysis based on GAR tertiles revealed that the 30-day, 90-day, and 365-day mortality rates were significantly lower in the Tertile 2, with statistically significant differences between the three groups (*P* < 0.001) (Fig. 2). This indicates that both high and low levels of GAR are associated with worse short-term and long-term outcomes in critically ill patients with AF.

### The association between GAR and patient clinical outcomes

Two Cox regression models were employed to investigate the independent influence of the GAR on mortality (Table 2), both unadjusted and adjusted for age, gender, race, AKI, CKD, HF, hypertension, cancer, stroke, WBC, hemoglobin, creatinine, SOFA score and OASIS score. Using the tertiles 2 as the reference in both models, heightened mortality risks were evident in the other two groups at 30 days, 90 days, and 365 days. In the unadjusted model, compared to the reference group (Tertile 2), the 30-day mortality risk for the third tertile was 2.42 (95% CI 1.83–3.21, *P* < 0.001), and for the first tertile, it was 1.69 (95% CI 1.25–2.28, *P* = 0.001). In the multivariate-adjusted model, the HR for the first tertile (reference: the second tertile, 1.00) was 1.53 (95% CI 1 ~ 1.83, *P* = 0.052), and for the third group, it was 2.56 (95% CI 1.99 ~ 3.54,* P* < 0.001), with a similar trend observed at 90 days and 365 days.

**Table 2:** The Cox proportional hazards model for all-cause mortality at 30 days, 90 days, and 365 days

GAR groups	Model I	*P*-value	Model II	*P*-value
30-day mortality risk				
Tertile1(1.97–14.03)	1.69 (1.25 ~ 2.28)	0.001	1.35 (1 ~ 1.83)	0.052
Tertile2 (14.04–16.54)	1(Ref)		1(Ref))	
Tertile3 (16.55–40.32)	2.42 (1.83 ~ 3.21)	< 0.001	2.65 (1.99 ~ 3.54)	< 0.001
90-day mortality risk				
Tertile1(1.97–14.03)	1.75 (1.38 ~ 2.21)	< 0.001	1.4 (1.1 ~ 1.78)	0.006
Tertile2 (14.04–16.54)	1(Ref)	< 0.001	1(Ref)	
Tertile3 (16.55–40.32)	1.90 (1.5 ~ 2.39)	< 0.001	2.13 (1.68 ~ 2.7)	< 0.001
365-day mortality risk				
Tertile1(1.97–14.03)	1.65 (1.36 ~ 1.99)	< 0.001	1.36 (1.12 ~ 1.65)	0.002
Tertile2 (14.04–16.54)	1(Ref)		1(Ref)	
Tertile3 (16.55–40.32)	1.51 (1.25 ~ 1.83)	< 0.001	1.7 (1.39 ~ 2.06)	< 0.001

Model I: Univariate model for groups stratified by GAR

Model II: Adjusted for age, gender, race, BMI, AKI, CKD, HF, hypertension, cancer, stroke, WBC, HGB, creatinine, serum lactate, SOFA score and OASIS score

*Ref* reference value

**Fig. 3:**
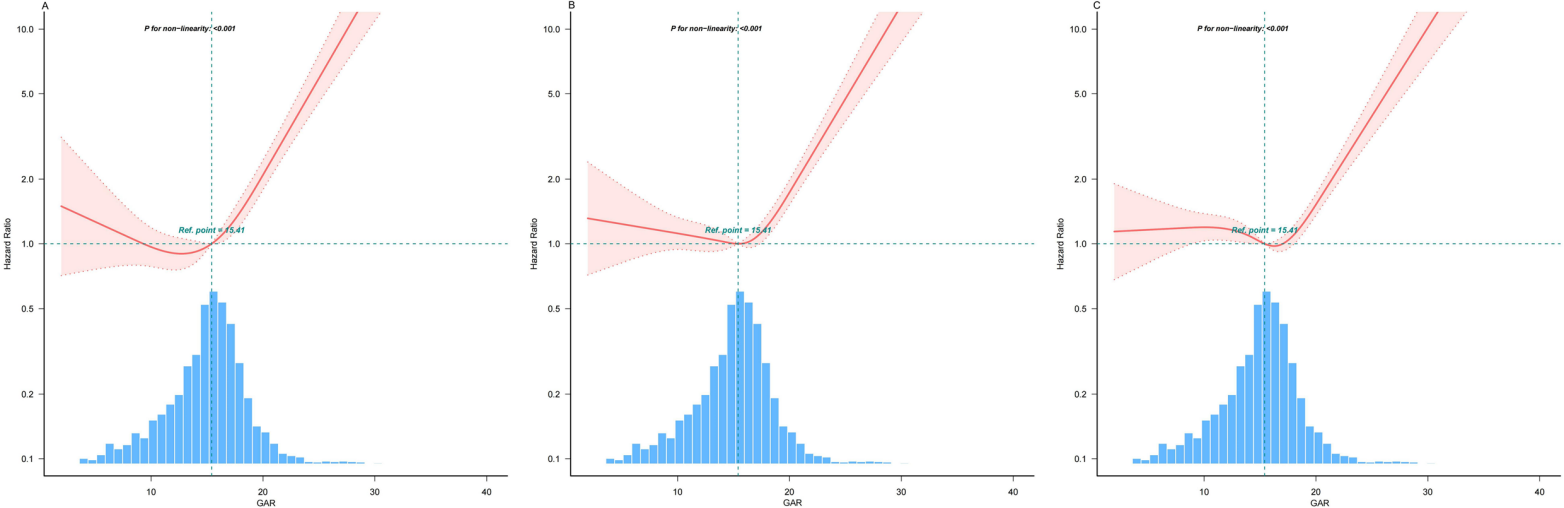
Restricted cubic spline curve analysis for GAR and mortality hazard ratio in critically ill patients with AF. **A** Restricted cubic spline curve for the mortality rate of patients within 30 days; **B** Restricted cubic spline curve for the mortality rate of patients within 90 days; **C** Restricted cubic spline curve for the mortality rate of patients within 365 days

The dose–response association between the GAR and 30-day, 90-day, and 365-day mortality rates is depicted in Fig. 3, revealing a nonlinear "J-shaped" relationship across all three time points (*P non-linear* < 0.001). Given the reliability of this nonlinear relationship, a threshold effect analysis was conducted, with the results presented in Table 3. The thresholds for mortality risk at 30-day, 90-day, and 365-day were determined to be 15.915, 17.363 and 18.214, respectively. Beyond these thresholds, the risk of mortality significantly increased with increasing GAR.

**Table 3:** Two-piecewise Cox proportional hazards model

	30-day mortality	*P* value	90-day mortality	*P* value	365-day mortality	*P* value
Threshold (K)”	15.915 (15.699,16.132)		17.363 (16.994,17.733)		18.214 (17.755,18.672)	
< K	0.959 (0.91,1.011)	0.1193	0.973 (0.939,1.008)	0.1271	0.973 (0.947,0.999)	0.0457
> K	1.272 (1.214,1.333)	< 0.001	1.258 (1.191,1.328)	< 0.001	1.228 (1.157,1.304)	< 0.001
Log-likelihood ratio test		< 0.001		< 0.001		< 0.001

### Subgroup analysis

Subgroup analyses were conducted for multiple characteristics includingage, gender, race, AKI, CKD, HF, hypertension, cancer, stroke and BMI. No interactions were found (*P* for interaction > 0.05), indicating robustness of the outcomes, as shown in Tables 4, 5, 6.

**Table 4:** Subgroup analysis of 30-day mortality among patients

Subgroup	Variable	Total	Event (%)	HR(95 CI)	*P* value	*P* for interaction
Age						0.348
< 65	Tertile1	233	17 (7.3)	1.93 (0.74 ~ 5.05)	0.18	
	Tertile2	233	6 (2.6)	1(Ref)		
	Tertile3	239	22 (9.2)	4.32 (1.68 ~ 11.08)	0.002	
	Trend test	705	45 (6.4)	1.53 (1.04 ~ 2.25)	0.029	
≥ 65	Tertile1	857	98 (11.4)	1.26 (0.92 ~ 1.74)	0.156	
	Tertile2	866	64 (7.4)	1(Ref)		
	Tertile3	857	137 (16)	2.45 (1.81 ~ 3.32)	< 0.001	
	Trend test	2580	299 (11.6)	1.43 (1.24 ~ 1.66)	< 0.001	
Gender						0.912
Female	Tertile1	457	58 (12.7)	1.39 (0.91 ~ 2.14)	0.129	
	Tertile2	424	35 (8.3)	1(Ref)		
	Tertile3	407	79 (19.4)	2.67 (1.78 ~ 3.99)	< 0.001	
	Trend test	1288	172 (13.4)	1.43 (1.18 ~ 1.73)	< 0.001	
Male	Tertile1	633	57 (9)	1.36 (0.88 ~ 2.09)	0.164	
	Tertile2	675	35 (5.2)	1(Ref)		
	Tertile3	689	80 (11.6)	2.79 (1.86 ~ 4.2)	< 0.001	
	Trend test	1997	172 (8.6)	1.48 (1.22 ~ 1.79)	< 0.001	
Race						0.96
White	Tertile1	692	65 (9.4)	1.46 (0.99 ~ 2.17)	0.059	
	Tertile2	765	42 (5.5)	1(Ref)		
	Tertile3	728	84 (11.5)	2.52 (1.73 ~ 3.69)	< 0.001	
	Trend test	2185	191 (8.7)	1.34 (1.12 ~ 1.62)	0.002	
Black	Tertile1	61	6 (9.8)	1.68 (0.38 ~ 7.55)	0.497	
	Tertile2	47	3 (6.4)	1(Ref)		
	Tertile3	41	11 (26.8)	6.25 (1.52 ~ 25.65)	0.011	
	Trend test	149	20 (13.4)	2.09 (1.11 ~ 3.94)	0.022	
Other	Tertile1	337	44 (13.1)	1.24 (0.75 ~ 2.05)	0.403	
	Tertile2	287	25 (8.7)	1(Ref)		
	Tertile3	327	64 (19.6)	2.52 (1.57 ~ 4.04)	< 0.001	
	Trend test	951	133 (14)	1.47 (1.19 ~ 1.81)	< 0.001	
BMI						0.104
≤ 24.9	Tertile1	275	38 (13.8)	1.16 (0.7 ~ 1.93)	0.558	
	Tertile2	259	27 (10.4)	1(Ref)		
	Tertile3	215	37 (17.2)	1.95 (1.15 ~ 3.28)	0.012	
	Trend test	749	102 (13.6)	1.28 (1 ~ 1.65)	0.051	
25–30	Tertile1	347	36 (10.4)	1.9 (1.06 ~ 3.39)	0.03	
	Tertile2	376	18 (4.8)	1(Ref)		
	Tertile3	373	47 (12.6)	3.5 (2 ~ 6.13)	< 0.001	
	Trend test	1096	101 (9.2)	1.4 (1.08 ~ 1.81)	0.01	
> 30	Tertile1	468	41 (8.8)	1.22 (0.73 ~ 2.03)	0.44	
	Tertile2	464	25 (5.4)	1(Ref)		
	Tertile3	508	75 (14.8)	3 (1.9 ~ 4.75)	< 0.001	
	Trend test	1440	141 (9.8)	1.66 (1.34 ~ 2.05)	< 0.001	
Hypertension						0.898
No	Tertile1	614	77 (12.5)	1.54 (1.03 ~ 2.31)	0.037	
	Tertile2	519	35 (6.7)	1(Ref)		
	Tertile3	495	76 (15.4)	2.64 (1.76 ~ 3.97)	< 0.001	
	Trend test	1628	188 (11.5)	1.31 (1.09 ~ 1.56)	0.003	
Yes	Tertile1	476	38 (8)	1.12 (0.7 ~ 1.8)	0.637	
	Tertile2	580	35 (6)	1(Ref)		
	Tertile3	601	83 (13.8)	2.61 (1.74 ~ 3.9)	< 0.001	
	Trend test	1657	156 (9.4)	1.64 (1.32 ~ 2.03)	< 0.001	
Heart failure						0.481
No	Tertile1	522	44 (8.4)	1.05 (0.67 ~ 1.63)	0.835	
	Tertile2	637	39 (6.1)	1(Ref)		
	Tertile3	698	94 (13.5)	2.44 (1.66 ~ 3.57)	< 0.001	
	Trend test	1857	177 (9.5)	1.61 (1.32 ~ 1.96)	< 0.001	
Yes	Tertile1	568	71 (12.5)	1.59 (1.04 ~ 2.45)	0.033	
	Tertile2	462	31 (6.7)	1(Ref)		
	Tertile3	398	65 (16.3)	2.97 (1.92 ~ 4.57)	< 0.001	
	Trend test	1428	167 (11.7)	1.36 (1.12 ~ 1.64)	0.002	
Cancer						0.358
No	Tertile1	894	82 (9.2)	1.2 (0.84 ~ 1.71)	0.328	
	Tertile2	901	51 (5.7)	1(Ref)		
	Tertile3	938	136 (14.5)	2.78 (2.01 ~ 3.85)	< 0.001	
	Trend test	2733	269 (9.8)	1.6 (1.37 ~ 1.86)	< 0.001	
Yes	Tertile1	196	33 (16.8)	1.79 (1 ~ 3.18)	0.049	
	Tertile2	198	19 (9.6)	1(Ref)		
	Tertile3	158	23 (14.6)	1.97 (1.05 ~ 3.7)	0.034	
	Trend test	552	75 (13.6)	1 (0.74 ~ 1.35)	0.977	
CKD						0.232
No	Tertile1	898	47 (5.2)	1(Ref)		
	Tertile2	769	73 (9.5)	1.54 (1.06 ~ 2.24)	0.022	
	Tertile3	910	121 (13.3)	3.03 (2.15 ~ 4.26)	< 0.001	
	Trend test	2577	241 (9.4)	1.49 (1.26 ~ 1.75)	< 0.001	
Yes	Tertile1	201	23 (11.4)	1(Ref)		
	Tertile2	321	42 (13.1)	1.06 (0.63 ~ 1.77)	0.839	
	Tertile3	186	38 (20.4)	1.82 (1.06 ~ 3.14)	0.03	
	Trend test	708	103 (14.5)	1.3 (1.02 ~ 1.65)	0.032	
AKI						0.44
No	Tertile1	663	39 (5.9)	1.19 (0.75 ~ 1.89)	0.457	
	Tertile2	809	36 (4.4)	1(Ref)		
	Tertile3	869	104 (12)	2.85 (1.95 ~ 4.18)	< 0.001	
	Trend test	2341	179 (7.6)	1.7 (1.39 ~ 2.09)	< 0.001	
Yes	Tertile1	427	76 (17.8)	1.55 (1.02 ~ 2.36)	0.038	
	Tertile2	290	34 (11.7)	1(Ref)		
	Tertile3	227	55 (24.2)	2.62 (1.69 ~ 4.07)	< 0.001	
	Trend test	944	165 (17.5)	1.26 (1.04 ~ 1.53)	0.02	
Stroke						0.654
No	Tertile1	954	94 (9.9)	1.32 (0.94 ~ 1.86)	0.109	
	Tertile2	915	53 (5.8)	1(Ref)		
	Tertile3	920	126 (13.7)	2.76 (1.99 ~ 3.82)	< 0.001	
	Trend test	2789	273 (9.8)	1.49 (1.28 ~ 1.73)	< 0.001	
Yes	Tertile1	136	21 (15.4)	1.51 (0.77 ~ 2.97)	0.235	
	Tertile2	184	17 (9.2)	1(Ref)		
	Tertile3	176	33 (18.8)	2.68 (1.43 ~ 5.01)	0.002	
	Trend test	496	71 (14.3)	1.41 (1.01 ~ 1.96)	0.041

**Table 5:** Subgroup analysis of 90-day mortality among patients

Subgroup	Variable	Total	Event (%)	HR (95CI)	*P* value	*P* for interaction
Age						0.334
< 65	Tertile1	233	23 (9.9)	1.88 (0.85 ~ 4.16)	0.117	
	Tertile2	233	9 (3.9)	1(Ref)		
	Tertile3	239	24 (10)	3.2 (1.44 ~ 7.11)	0.004	
	Trend test	705	56 (7.9)	1.3 (0.93 ~ 1.83)	0.13	
≥ 65	Tertile1	857	164 (19.1)	1.34 (1.04 ~ 1.73)	0.022	
	Tertile2	866	102 (11.8)	1(Ref)		
	Tertile3	857	172 (20.1)	2.02 (1.58 ~ 2.59)	< 0.001	
	Trend test	2580	438 (17)	1.23 (1.09 ~ 1.39)	0.001	
Gender						0.939
Female	Tertile1	457	96 (21)	1.39 (1 ~ 1.94)	0.052	
	Tertile2	424	59 (13.9)	1(Ref)		
	Tertile3	407	102 (25.1)	2.16 (1.56 ~ 2.99)	< 0.001	
	Trend test	1288	257 (20)	1.26 (1.07 ~ 1.47)	0.005	
Male	Tertile1	633	91 (14.4)	1.48 (1.04 ~ 2.09)	0.028	
	Tertile2	675	52 (7.7)	1(Ref)		
	Tertile3	689	94 (13.6)	2.27 (1.6 ~ 3.22)	< 0.001	
	Trend test	1997	237 (11.9)	1.24 (1.05 ~ 1.46)	0.009	
Race						0.855
White	Tertile1	692	115 (16.6)	1.63 (1.2 ~ 2.21)	0.002	
	Tertile2	765	68 (8.9)	1(Ref)		
	Tertile3	728	109 (15)	2.12 (1.55 ~ 2.88)	< 0.001	
	Trend test	2185	292 (13.4)	1.13 (0.97 ~ 1.31)	0.104	
Black	Tertile1	61	11 (18)	1.14 (0.4 ~ 3.25)	0.803	
	Tertile2	47	6 (12.8)	1(Ref)		
	Tertile3	41	14 (34.1)	3.18 (1.12 ~ 9.05)	0.03	
	Trend test	149	31 (20.8)	1.67 (1.06 ~ 2.66)	0.029	
Other	Tertile1	337	61 (18.1)	1.16 (0.77 ~ 1.77)	0.477	
	Tertile2	287	37 (12.9)	1(Ref)		
	Tertile3	327	73 (22.3)	1.99 (1.33 ~ 2.99)	0.001	
	Trend test	951	171 (18)	1.32 (1.1 ~ 1.59)	0.003	
BMI						0.365
≤ 24.9	Tertile1	275	73 (26.5)	1.58 (1.06 ~ 2.35)	0.025	
	Tertile2	259	39 (15.1)	1(Ref)		
	Tertile3	215	53 (24.7)	1.97 (1.28 ~ 3.02)	0.002	
	Trend test	749	165 (22)	1.08 (0.89 ~ 1.32)	0.438	
25–30	Tertile1	347	53 (15.3)	1.6 (1.02 ~ 2.5)	0.04	
	Tertile2	376	33 (8.8)	1(Ref)		
	Tertile3	373	55 (14.7)	2.35 (1.5 ~ 3.68)	< 0.001	
	Trend test	1096	141 (12.9)	1.22 (0.98 ~ 1.51)	0.08	
> 30	Tertile1	468	61 (13)	1.18 (0.78 ~ 1.78)	0.432	
	Tertile2	464	39 (8.4)	1(Ref)		
	Tertile3	508	88 (17.3)	2.28 (1.56 ~ 3.35)	< 0.001	
	Trend test	1440	188 (13.1)	1.43 (1.2 ~ 1.72)	< 0.001	
Hypertension						0.527
No	Tertile1	614	123 (20)	1.41 (1.03 ~ 1.92)	0.03	
	Tertile2	519	63 (12.1)	1(Ref)		
	Tertile3	495	99 (20)	1.99 (1.44 ~ 2.75)	< 0.001	
	Trend test	1628	285 (17.5)	1.17 (1.01 ~ 1.35)	0.037	
Yes	Tertile1	476	64 (13.4)	1.38 (0.94 ~ 2.03)	0.098	
	Tertile2	580	48 (8.3)	1(Ref)		
	Tertile3	601	97 (16.1)	2.21 (1.55 ~ 3.14)	< 0.001	
	Trend test	1657	209 (12.6)	1.32 (1.1 ~ 1.57)	0.003	
Heart failure						0.935
No	Tertile1	522	79 (15.1)	1.32 (0.92 ~ 1.88)	0.131	
	Tertile2	637	54 (8.5)	1(Ref)		
	Tertile3	698	114 (16.3)	2.15 (1.54 ~ 2.99)	< 0.001	
	Trend test	1857	247 (13.3)	1.32 (1.12 ~ 1.55)	0.001	
Yes	Tertile1	568	108 (19)	1.41 (1.01 ~ 1.95)	0.041	
	Tertile2	462	57 (12.3)	1(Ref)		
	Tertile3	398	82 (20.6)	2.11 (1.5 ~ 2.98)	< 0.001	
	Trend test	1428	247 (17.3)	1.2 (1.02 ~ 1.41)	0.025	
Cancer						0.265
No	Tertile1	894	135 (15.1)	1.21 (0.91 ~ 1.6)	0.186	
	Tertile2	901	84 (9.3)	1(Ref)		
	Tertile3	938	171 (18.2)	2.22 (1.7 ~ 2.9)	< 0.001	
	Trend test	2733	390 (14.3)	1.38 (1.22 ~ 1.57)	< 0.001	
Yes	Tertile1	196	52 (26.5)	2.07 (1.28 ~ 3.34)	0.003	
	Tertile2	198	27 (13.6)	1(Ref)		
	Tertile3	158	25 (15.8)	1.47 (0.83 ~ 2.59)	0.183	
	Trend test	552	104 (18.8)	0.78 (0.6 ~ 1.01)	0.064	
CKD						0.429
No	Tertile1	769	119 (15.5)	1.64 (1.22 ~ 2.2)	0.001	
	Tertile2	898	74 (8.2)	1(Ref)		
	Tertile3	910	142 (15.6)	2.35 (1.77 ~ 3.13)	< 0.001	
	Trend test	2577	335 (13)	1.22 (1.06 ~ 1.4)	0.005	
Yes	Tertile1	321	68 (21.2)	1.07 (0.71 ~ 1.61)	0.738	
	Tertile2	201	37 (18.4)	1(Ref)		
	Tertile3	186	54 (29)	1.73 (1.12 ~ 2.67)	0.014	
	Trend test	708	159 (22.5)	1.25 (1.03 ~ 1.52)	0.023	
AKI						0.858
No	Tertile1	663	77 (11.6)	1.4 (0.99 ~ 1.99)	0.057	
	Tertile2	809	58 (7.2)	1(Ref)		
	Tertile3	869	123 (14.2)	2.17 (1.59 ~ 2.98)	< 0.001	
	Trend test	2341	258 (11)	1.3 (1.11 ~ 1.53)	0.001	
Yes	Tertile1	427	110 (25.8)	1.47 (1.05 ~ 2.06)	0.025	
	Tertile2	290	53 (18.3)	1(Ref)		
	Tertile3	227	73 (32.2)	2.3 (1.59 ~ 3.31)	< 0.001	
	Trend test	944	236 (25)	1.2 (1.02 ~ 1.42)	0.027	
Stroke						0.557
No	Tertile1	954	153 (16)	1.41 (1.08 ~ 1.86)	0.013	
	Tertile2	915	83 (9.1)	1(Ref)		
	Tertile3	920	153 (16.6)	2.24 (1.71 ~ 2.93)	< 0.001	
	Trend test	2789	389 (13.9)	1.26 (1.11 ~ 1.43)	< 0.001	
Yes	Tertile1	136	34 (25)	1.53 (0.9 ~ 2.58)	0.115	
	Tertile2	184	28 (15.2)	1(Ref)		
	Tertile3	176	43 (24.4)	2.11 (1.27 ~ 3.52)	0.004	
	Trend test	496	105 (21.2)	1.2 (0.92 ~ 1.56)	0.18

**Table 6:** Subgroup analysis of 365-day mortality among patients

Subgroup	Variable	Total	Event (%)	HR (95CI)	*P* value	*P* for interaction
Age						0.548
< 65	Tertile1	233	37 (15.9)	1.45 (0.84 ~ 2.51)	0.187	
	Tertile2	233	21 (9)	1(Ref)		
	Tertile3	239	27 (11.3)	1.62 (0.9 ~ 2.94)	0.11	
	Trend test	705	85 (12.1)	1.03 (0.78 ~ 1.36)	0.819	
≥ 65	Tertile1	857	239 (27.9)	1.35 (1.1 ~ 1.66)	0.004	
	Tertile2	866	157 (18.1)	1(Ref)		
	Tertile3	857	222 (25.9)	1.72 (1.4 ~ 2.12)	< 0.001	
	Trend test	2580	618 (24)	1.13 (1.02 ~ 1.25)	0.022	
Gender						0.795
Female	Tertile1	457	136 (29.8)	1.29 (0.98 ~ 1.69)	0.068	
	Tertile2	424	92 (21.7)	1(Ref)		
	Tertile3	407	124 (30.5)	1.68 (1.28 ~ 2.21)	< 0.001	
	Trend test	1288	352 (27.3)	1.14 (0.99 ~ 1.3)	0.061	
Male	Tertile1	633	140 (22.1)	1.48 (1.13 ~ 1.95)	0.005	
	Tertile2	675	86 (12.7)	1(Ref)		
	Tertile3	689	125 (18.1)	1.82 (1.38 ~ 2.41)	< 0.001	
	Trend test	1997	351 (17.6)	1.1 (0.96 ~ 1.26)	0.169	
Race						0.784
White	Tertile1	692	184 (26.6)	1.51 (1.19 ~ 1.9)	0.001	
	Tertile2	765	120 (15.7)	1(Ref)		
	Tertile3	728	148 (20.3)	1.58 (1.24 ~ 2.02)	< 0.001	
	Trend test	2185	452 (20.7)	1.01 (0.89 ~ 1.14)	0.895	
Black	Tertile1	61	18 (29.5)	1.4 (0.57 ~ 3.43)	0.465	
	Tertile2	47	8 (17)	1(Ref)		
	Tertile3	41	18 (43.9)	2.91 (1.17 ~ 7.22)	0.021	
	Trend test	149	44 (29.5)	1.44 (0.98 ~ 2.1)	0.062	
Other	Tertile1	337	74 (22)	1.08 (0.75 ~ 1.57)	0.675	
	Tertile2	287	50 (17.4)	1(Ref)		
	Tertile3	327	83 (25.4)	1.71 (1.19 ~ 2.45)	0.004	
	Trend test	951	207 (21.8)	1.26 (1.07 ~ 1.5)	0.007	
BMI						0.481
≤ 24.9	Tertile1	275	96 (34.9)	1.45 (1.03 ~ 2.02)	0.031	
	Tertile2	259	58 (22.4)	1(Ref)		
	Tertile3	215	64 (29.8)	1.61 (1.11 ~ 2.32)	0.011	
	Trend test	749	218 (29.1)	1.02 (0.86 ~ 1.22)	0.789	
25–30	Tertile1	347	83 (23.9)	1.44 (1.01 ~ 2.04)	0.041	
	Tertile2	376	56 (14.9)	1(Ref)		
	Tertile3	373	76 (20.4)	1.75 (1.23 ~ 2.5)	0.002	
	Trend test	1096	215 (19.6)	1.1 (0.92 ~ 1.31)	0.294	
> 30	Tertile1	468	97 (20.7)	1.2 (0.86 ~ 1.66)	0.282	
	Tertile2	464	64 (13.8)	1(Ref)		
	Tertile3	508	109 (21.5)	1.76 (1.29 ~ 2.41)	< 0.001	
	Trend test	1440	270 (18.8)	1.23 (1.05 ~ 1.43)	0.008	
Hypertension						0.3
No	Tertile1	614	181 (29.5)	1.29 (1.01 ~ 1.65)	0.041	
	Tertile2	519	106 (20.4)	1(Ref)		
	Tertile3	495	128 (25.9)	1.52 (1.17 ~ 1.98)	0.002	
	Trend test	1628	415 (25.5)	1.07 (0.94 ~ 1.21)	0.301	
Yes	Tertile1	476	95 (20)	1.45 (1.06 ~ 1.98)	0.022	
	Tertile2	580	72 (12.4)	1(Ref)		
	Tertile3	601	121 (20.1)	1.85 (1.38 ~ 2.49)	< 0.001	
	Trend test	1657	288 (17.4)	1.16 (1 ~ 1.35)	0.056	
Heart failure						0.947
No	Tertile1	522	120 (23)	1.51 (1.12 ~ 2.02)	0.006	
	Tertile2	637	78 (12.2)	1(Ref)		
	Tertile3	698	133 (19.1)	1.75 (1.31 ~ 2.33)	< 0.001	
	Trend test	1857	331 (17.8)	1.09 (0.95 ~ 1.25)	0.237	
Yes	Tertile1	568	156 (27.5)	1.22 (0.95 ~ 1.57)	0.127	
	Tertile2	462	100 (21.6)	1(Ref)		
	Tertile3	398	116 (29.1)	1.67 (1.27 ~ 2.19)	< 0.001	
	Trend test	1428	372 (26.1)	1.15 (1.01 ~ 1.31)	0.035	
Cancer						0.515
No	Tertile1	894	207 (23.2)	1.23 (0.98 ~ 1.54)	0.069	
	Tertile2	901	136 (15.1)	1(Ref)		
	Tertile3	938	209 (22.3)	1.73 (1.39 ~ 2.15)	< 0.001	
	Trend test	2733	552 (20.2)	1.19 (1.07 ~ 1.33)	0.001	
Yes	Tertile1	196	69 (35.2)	1.85 (1.25 ~ 2.75)	0.002	
	Tertile2	198	42 (21.2)	1(Ref)		
	Tertile3	158	40 (25.3)	1.49 (0.95 ~ 2.33)	0.083	
	Trend test	552	151 (27.4)	0.85 (0.68 ~ 1.05)	0.137	
CKD						0.516
No	Tertile1	769	171 (22.2)	1.55 (1.23 ~ 1.97)	< 0.001	
	Tertile2	898	119 (13.3)	1(Ref)		
	Tertile3	910	180 (19.8)	1.84 (1.45 ~ 2.32)	< 0.001	
	Trend test	2577	470 (18.2)	1.09 (0.97 ~ 1.23)	0.145	
Yes	Tertile1	321	105 (32.7)	1.09 (0.79 ~ 1.52)	0.587	
	Tertile2	201	59 (29.4)	1(Ref)		
	Tertile3	186	69 (37.1)	1.45 (1.01 ~ 2.08)	0.043	
	Trend test	708	233 (32.9)	1.13 (0.96 ~ 1.33)	0.131	
AKI						0.403
No	Tertile1	663	129 (19.5)	1.43 (1.09 ~ 1.86)	0.01	
	Tertile2	809	100 (12.4)	1(Ref)		
	Tertile3	869	155 (17.8)	1.59 (1.24 ~ 2.05)	< 0.001	
	Trend test	2341	384 (16.4)	1.08 (0.95 ~ 1.22)	0.269	
Yes	Tertile1	427	147 (34.4)	1.35 (1.02 ~ 1.79)	0.036	
	Tertile2	290	78 (26.9)	1(Ref)		
	Tertile3	227	94 (41.4)	2 (1.47 ~ 2.72)	< 0.001	
	Trend test	944	319 (33.8)	1.17 (1.02 ~ 1.35)	0.027	
Stroke						0.951
No	Tertile1	954	226 (23.7)	1.34 (1.08 ~ 1.67)	0.008	
	Tertile2	915	136 (14.9)	1(Ref)		
	Tertile3	920	192 (20.9)	1.7 (1.36 ~ 2.12)	< 0.001	
	Trend test	2789	554 (19.9)	1.11 (1 ~ 1.24)	0.045	
Yes	Tertile1	136	50 (36.8)	1.52 (0.99 ~ 2.33)	0.054	
	Tertile2	184	42 (22.8)	1(Ref)		
	Tertile3	176	57 (32.4)	1.84 (1.21 ~ 2.81)	0.004	
	Trend test	496	149 (30)	1.11 (0.89 ~ 1.38)	0.359	

## Discussion

This study explored the relationship between the GAR, a representative marker of stress-induced hyperglycemia, and the risk of mortality in critically ill patients with AF. We observed that both excessively high and low levels of the GAR are associated with increased risks of short-term and long-term mortality. This relationship persisted even after adjusting for multiple confounding factors. Based on the restricted cubic splines (RCS) curve, a “J-shaped” relationship was established, and threshold analysis of continuous variables was employed to explore the inflection points of the GAR at various survival time points. Additionally, subgroup analyses revealed no interaction effects.

The occurrence of AF is associated with the cardiac electrophysiology, defects in specific molecular pathways, and structural changes in the left atrium [13]. Improvements in the prognosis of AF patients primarily focus on heart rate control, anticoagulation, and stroke prevention [14]. Although catheter ablation can cure AF, it often accompanies uncontrollable recurrence postoperatively. Current research has also demonstrated that preventing nicotinamide adenine dinucleotide (NAD) depletion and subsequent myocardial cell dysfunction, inhibiting inflammatory compounds, and regulating calcium ion homeostasis can improve the prognosis of AF [13].

In fact, as mentioned earlier, myocardial metabolism primarily relies on fatty acids rather than glucose. During periods of stress hyperglycemia, activation of adrenergic responses, increased inflammation and oxidative stress, formation of glycation end products due to high glucose levels, and myocardial dysfunction caused by vigorous glucose metabolism in the myocardium may occur [15]. Additionally, epicardial adipose tissue (EAT) [16] is considered relevant to AF. Against the backdrop of AF, the inflammatory response in EAT can induce fibrosis in atrial myocytes and disrupt neurohormonal factors through regional secretion, accelerating the progression of heart failure. A randomized controlled trial has shown that SGLT-2 inhibition selectively reduces glucose uptake in EAT among patients with type 2 diabetes, decreasing EAT inflammation and thereby enhancing myocardial blood flow to provide a protective effect [16]. Meanwhile, metabolic abnormalities induced by stress hyperglycemia may promote the onset and persistence of AF by regulating atrial substrates, disrupting myocardial energy metabolism and electrical remodeling, and modulating myocardial ion channels, ultimately leading to poor prognosis in AF [17–20]. Epidemiologically, the impact of stress hyperglycemia on new-onset AF following myocardial infarction has been studied utilizing the stress hyperglycemia ratio (SHR) [21]. Some studies have revealed multifaceted associations between insulin resistance and AF prognosis, post-ablation recurrence, and incident cases in the general populace [22–25]. Additionally, Terauchi et al. proposed a correlation between HbA1c levels ≥ 8.0% and heightened all-cause mortality risk among AF patients [22]. Although stress-induced hyperglycemia and AF are considered to be related, evidence is lacking regarding the impact of stress hyperglycemia on the prognosis of AF.

Critically ill patients are particularly susceptible to stress-induced hyperglycemia, a phenomenon more prevalent among them compared to individuals in general wards and healthy populations [4, 5]. The intricate interplay of acute systemic inflammation, hormonal fluctuations, and cytokine dysregulation precipitates excessive hepatic glucose secretion, lipid peroxidation, gluconeogenesis, and heightened insulin resistance, collectively contributing to the development of stress-induced hyperglycemia [5, 26–28]. Notably, diverse metrics serve as proxies for stress-induced hyperglycemia [29]. Our study found robust J-shaped curve outcomes for both short-term and long-term prognosis in critically ill patients with AF when stress-induced hyperglycemia was represented by the GAR. Moreover, no matter which time point was considered as the observed outcome, the risk of mortality increased with the increase in GAR beyond a certain threshold. Additionally, when the 365-day mortality risk was considered as the study outcome, GAR exhibited a protective factor as it decreases below the threshold. These findings hold substantial clinical significance, particularly given the ongoing debate surrounding glycemic management in critically ill patients [30–34]. A recent article in The Lancet Diabetes & Endocrinology underscored the importance of glycemic management in both diabetic and non-diabetic critically ill populations [35].

Considering the high prevalence of AF in ICU settings [2], coupled with the close association between AF and stress-induced hyperglycemia, our study provides valuable insights for guiding future glycemic targets in critically ill patients with AF. Additionally, it aids in identifying critically ill AF patients at high risk of mortality.

### Limitations

This is a retrospective study and cannot establish causality. The lowest blood glucose value may not actually represent fasting blood glucose. Additionally, glycated hemoglobin has limitations and is influenced by factors such as ethnicity, blood transfusions, certain hemoglobinopathies, hemolytic anemia, post-splenectomy status, polycythemia, and even iron-deficiency anemia. According to previous studies, blood lipids and are significant confounding factors for AF. However, due to over 30% missing data for these indicators, they had to be excluded, which may impact the study results.

## Conclusion

The GAR levels exhibited a "J-shaped" linear correlation with both short-term and long-term outcomes in critically ill AF patients. Elevated or reduced GAR levels may indicate adverse prognoses for these patients. This conclusion provides a basis for glucose management in critically ill AF patients.

## Data Availability

Data used can be obtained upon a reasonable request to the corresponding author.

## Abbreviations

GAR: Glucose-to-glycated hemoglobin ratio
AF: Atrial fibrillation
HbA1c: Glycosylated hemoglobin
ICU: Intensive care unit
MIMIC-IV: Medical Information Mart for Intensive Care IV
AMI: Acute myocardial infarction
HF: Heart failure
CKD: Chronic kidney disease
WBC: White blood cells
HGB: Hemoglobin
SOFA: Sepsis-organ failure assessment score
OASIS: Oxford acute severity of illness score
RCS: Restricted cubic spline
HR: Hazard ratio
SHR: Stress hyperglycemia ratio
EAT: Epicardial adipose tissue
